# Clinical and hematological factors associated with neurological involvement in spinal tuberculosis: a retrospective study

**DOI:** 10.3389/fcimb.2026.1785055

**Published:** 2026-05-01

**Authors:** Linan Wang, Xingyu Duan, Yuxin Gao, Xueyan Jing, Jiong Wang, Lijun Feng, Hekun Liu, Zhiyun Shi, Ningkui Niu

**Affiliations:** 1 Department of Orthopedics, General Hospital of Ningxia Medical University, Yinchuan, China; 2 The First Clinical Medical College of Ningxia Medical University, Yinchuan, China; 3 Medical Experiment Center, General Hospital of Ningxia Medical University, Yinchuan, China; 4 Ningxia Key Laboratory of Clinical and Pathogenic Microbiology, General Hospital of Ningxia Medical University, Yinchuan, China; 5 Research Center for Prevention and Control of Bone and Joint Tuberculosis, General Hospital of Ningxia Medical University, Yinchuan, China

**Keywords:** lymphocyte percentage (LYM), mixed cell percentage (MXD), neurological involvement, predictive model, spinal tuberculosis

## Abstract

**Background:**

The association between peripheral blood immune-inflammatory markers and neurological involvement in patients with spinal tuberculosis, and to develop a predictive model for early risk assessment. Spinal tuberculosis with neurological impairment is a major cause of disability, yet simple, cost-effective early warning indicators remain lacking. Peripheral blood immune-inflammatory markers are valuable in infectious disease prognostication, but their predictive role in spinal tuberculosis neurological involvement remains unclear.

**Methods:**

A retrospective study was conducted on 294 patients with spinal tuberculosis who underwent surgical treatment in the Department of Orthopedics, General Hospital of Ningxia Medical University, between December 2019 and December 2024. Patients were stratified into two groups: the uncomplicated spinal tuberculosis group (n=194, ASIA grade E) and the neurological involvement group (n=100, ASIA grades A–D). Fasting venous blood test results collected at the initial presentation were analyzed. Immune-inflammatory markers, including lymphocyte percentage (LYM), mixed cell percentage (MXD), and platelet-to-lymphocyte ratio (PLR), were compared between the two groups. Univariate and multivariate logistic regression analyses were performed to identify influencing factors, followed by the establishment of a predictive model. Receiver operating characteristic (ROC) curves and a nomogram were constructed to evaluate the diagnostic efficacy of the model.

**Results:**

The neurological involvement group exhibited a significantly lower LYM [23.150% (16.900, 29.525) vs. 26.150% (19.750, 32.900)], and significantly higher MXD [9.000% (7.375, 10.200) vs. 8.100% (6.725, 9.900)] and PLR compared with the uncomplicated group (*P* < 0.05). Multivariate analysis revealed that LYM (odds ratio [OR]=0.961, 95% confidence interval [CI]: 0.935–0.989) and MXD (OR = 1.107, 95% CI: 1.013–1.209) were independent predictors of neurological involvement in spinal tuberculosis. Specifically, each 1% increase in LYM was associated with a 3.9% reduction in the risk of neurological impairment, whereas each 1% increase in MXD correlated with a 10.7% increase in risk. The combined predictive model achieved an AUC of 0.803 (95% CI: 0.749–0.857), with a sensitivity of 70.0% and specificity of 80.9%. The calibration curve confirmed good model fit (χ²=4.215, P = 0.837). In addition, the Brier score was 0.185, indicating favorable overall accuracy of the probabilistic predictions.

**Conclusion:**

Decreased peripheral blood LYM and increased MXD are independent risk factors for neurological involvement in spinal tuberculosis. The combined predictive model shows favorable diagnostic efficacy and calibration. The nomogram is a simple, economical clinical tool for early high-risk patient identification, guiding individualized treatment decisions.

## 1 Introduction

Spinal tuberculosis accounts for more than 50% of all osteoarticular tuberculosis cases, and nearly half of these patients develop neurological impairment, which represents one of the predominant causes of permanent disability (Ruparel et al., 2022; Gan et al., 2024). However, simple and cost-effective early warning indicators remain clinically scarce, impeding the timely identification of high-risk individuals (Badhiwala et al., 2021).

Existing research has predominantly focused on local imaging features such as spinal canal encroachment and kyphotic deformity (Han et al., 2023; Subramanian et al., 2025), with insufficient attention paid to the host’s systemic immune-inflammatory status. Mycobacterium tuberculosis infection can induce lymphocyte subset imbalance, proinflammatory cytokine release, and platelet activation. These pathological processes may exacerbate spinal cord edema and ischemia, thereby facilitating the progression of neurological dysfunction (Vu et al., 2024; Boom et al., 2021). Notably, peripheral blood immune-inflammatory markers—including lymphocyte percentage (LYM), mixed cell percentage (MXD), and platelet-to-lymphocyte ratio (PLR)—have been extensively applied in prognostic assessment of malignant tumors and infectious diseases (Worapongpaiboon et al., 2025; Wu et al., 2025; Li et al., 2023; Huang et al., 2022), yet their predictive value for neurological involvement in spinal tuberculosis remains elusive. Furthermore, previous studies have mostly relied on small sample sizes and univariate analyses (Mittal et al., 2021; Rai et al., 2022), failing to adequately control confounding variables, which has led to conflicting conclusions.

In this study, we conducted a retrospective analysis of 294 patients with spinal tuberculosis and employed multivariate logistic regression models to systematically investigate the correlations between peripheral blood immune-inflammatory markers and neurological involvement. The primary objective was to provide clinicians with an easily accessible predictive tool that can guide individualized treatment strategies and improve neurological functional outcomes in affected patients.

## 2 Materials and methods

### 2.1 Study participants

**Figure 1:**
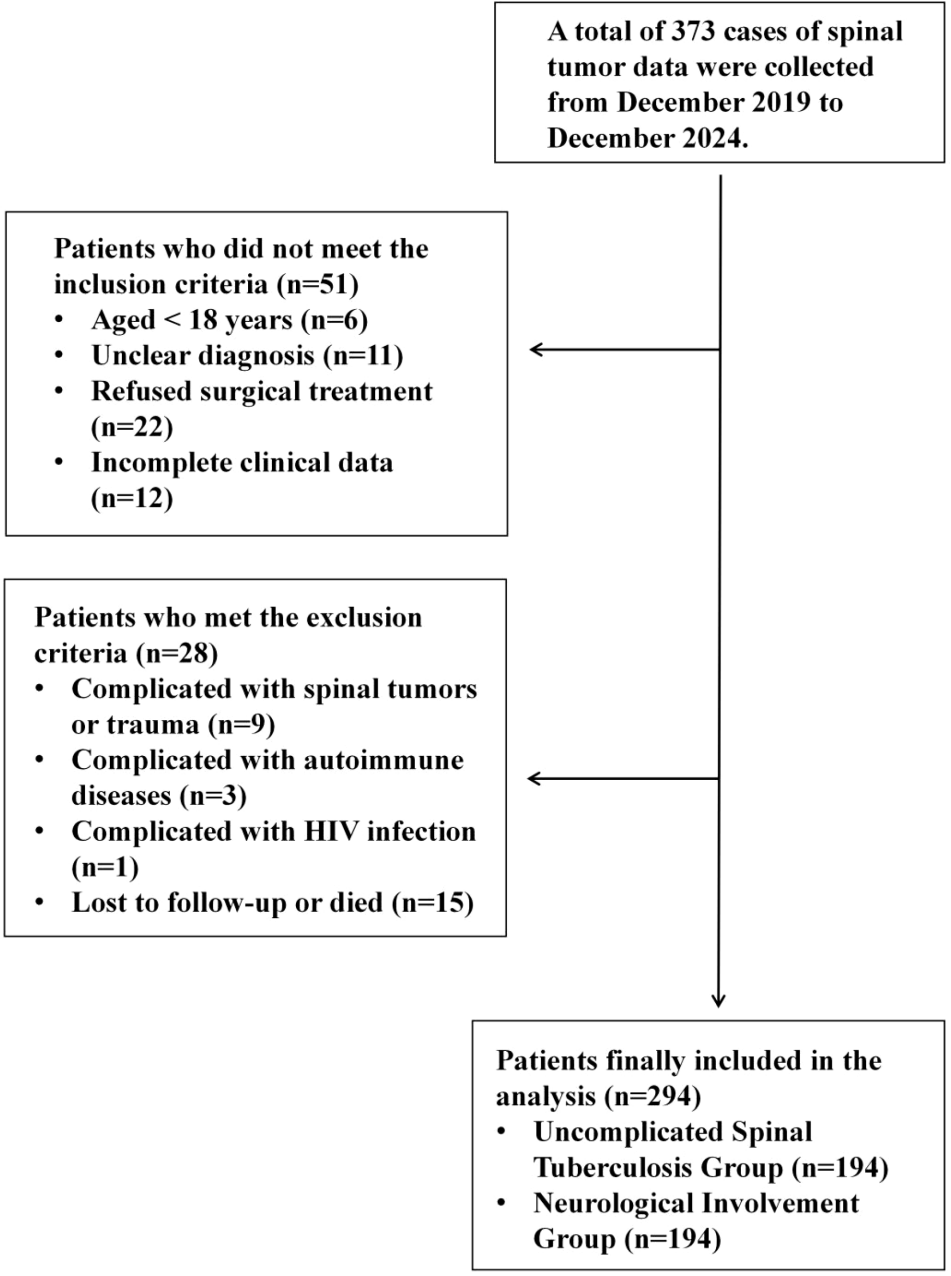
Flowchart of patient inclusion in the study.

This study was designed as a single-center retrospective cohort study, enrolling 294 patients diagnosed with spinal tuberculosis who underwent surgical treatment in the Department of Orthopedics, General Hospital of Ningxia Medical University, between December 2019 and December 2024. The detailed data screening process is illustrated in **Figure 1**.

### 2.2 Inclusion criteria

①Confirmed diagnosis of spinal tuberculosis, defined as meeting at least two of the following three criteria: (1) clinical manifestations including tuberculous toxic symptoms and local pain; (2) imaging findings (X-ray, computed tomography [CT] or magnetic resonance imaging [MRI]) demonstrating vertebral bone destruction, intervertebral space stenosis or obliteration, and paravertebral abscess formation; (3) laboratory evidence including positive tuberculin skin test, positive tuberculosis infection T-cell assay, or positive Mycobacterium tuberculosis culture of abscess pus.

②Aged 18 years or older.

③Complete preoperative data on neurological function assessment and laboratory examinations.

④Received standardized anti-tuberculosis chemotherapy combined with surgical intervention.

### 2.3 Exclusion criteria

①Concurrent spinal tumors, traumatic injuries, degenerative spinal disorders, or other spinal cord lesions.

②Complicated with autoimmune diseases (e.g., rheumatoid arthritis, systemic lupus erythematosus) or administration of glucocorticoids or immunosuppressants within the previous 3 months.

③Human immunodeficiency virus (HIV) infection or other severe immunodeficiency diseases.

④Incomplete clinical data, loss to follow-up, or deceased patients.

Patients were stratified into two groups based on their preoperative neurological status: the uncomplicated spinal tuberculosis group (n=194), consisting of patients without definite neurological deficit symptoms and classified as American Spinal Injury Association (ASIA) grade E; and the neurological involvement group (n=100), comprising patients presenting with neurological symptoms such as lower extremity paresthesia, muscle weakness, and bowel or bladder dysfunction, with ASIA grades ranging from A to D. This study was approved by the Institutional Review Board of the hospital (Approval No. KYLL20220133). Given its retrospective design, the requirement for informed consent was waived. In addition to the binary classification (ASIA grades A–D vs. grade E), a subgroup analysis was further conducted within the neurological involvement group. Patients were stratified into severe neurological impairment (ASIA grades A–B) and mild-to-moderate impairment (ASIA grades C–D) to explore the association between immune-inflammatory markers and the severity of neurological injury.

### 2.4 Data collection

Data were retrieved from the hospital’s Hospital Information System (HIS) and Picture Archiving and Communication System (PACS).

1. General data: Age, sex, body mass index (BMI), smoking history, diabetes mellitus history, tuberculosis history, symptom duration, and treatment delay time (defined as the interval from the onset of initial symptoms to the administration of surgical treatment).
2. Laboratory examinations: Results of the first fasting venous blood tests collected after admission were obtained. The analyzed variables included inflammatory markers (WBC, NEUT, LYM, MXD, EOS), absolute cell counts (NEUTcount, LYMcount, MXDcount, EOScount), hematological parameters (RBC, HGB, PLT), derived indices (PLR), and biochemical indicators (ALB, CRP, ESR, liver function parameters including AST, ALT, and ALP).
3. Neurological function assessment: The ASIA impairment scale (Mittal et al., 2021; Rai et al., 2022) was adopted for evaluation. Assessments were independently conducted by two orthopedic surgeons with over 10 years of clinical experience within 24 hours before surgery. The evaluation items included pinprick and light touch sensation scores at key sensory points, manual muscle testing scores (0–5 grades) of key muscles, as well as assessment of voluntary anal sphincter contraction and deep sensation. In cases of discrepant evaluations between the two surgeons, a chief physician (with more than 20 years of clinical practice) in the department performed a review and made the final determination. Patients classified as ASIA grades A–D were assigned to the neurological involvement group, while those with ASIA grade E were enrolled in the uncomplicated spinal tuberculosis group.

Radiological parameters were not included in this study because the primary objective was to develop a simple and easily applicable prediction model based on routinely available laboratory indicators, particularly for use in resource-limited settings.

### 2.5 Statistical analysis methods

All statistical analyses were performed using SPSS 26.0 software (IBM Corp., Armonk, NY, USA). Normality testing (Kolmogorov-Smirnov test) was first conducted for continuous variables. Variables conforming to a normal distribution were expressed as mean ± standard deviation (x¯ ± s), and comparisons between groups were performed using the independent-samples t-test. Variables with a non-normal distribution were presented as median (interquartile range) [M (Q_1_, Q_3_)], and the Mann-Whitney *U* test was used for intergroup comparisons. Categorical variables were expressed as frequency (percentage) [n (%)], and intergroup comparisons were analyzed using the Chi-square test or Fisher’s exact test. Univariate logistic regression analysis was performed to identify factors associated with neurological involvement. Variables with a *P*-value < 0.10 in the univariate analysis were further included in the multivariate logistic regression model. Stepwise forward selection (Forward: LR) was employed to screen for independent influencing factors, with the significance level for variable entry set at 0.05 and for variable removal set at 0.10. Odds ratios (ORs) and their corresponding 95% confidence intervals (CIs) were reported. The Hosmer-Lemeshow test was used to assess the goodness-of-fit of the model, with a *P*-value > 0.05 indicating a satisfactory fit. All tests were two-tailed, and a *P*-value < 0.05 was considered statistically significant. To further evaluate model performance, the Brier score was calculated to assess the overall accuracy of probabilistic predictions. Additionally, multicollinearity among candidate variables was assessed using the Variance Inflation Factor (VIF), with a VIF value < 5 considered indicative of acceptable collinearity. All statistical analyses were reviewed and verified to ensure methodological accuracy and robustness.

This study was reported in accordance with the Transparent Reporting of a Multivariable Prediction Model for Individual Prognosis or Diagnosis (TRIPOD) guidelines.

## 3 Results

A total of 294 patients with spinal tuberculosis were enrolled in this study, including 194 cases (66.0%) in the uncomplicated spinal tuberculosis group and 100 cases (34.0%) in the neurological involvement group. The baseline characteristics of the patients are presented in **Table 1**. No statistically significant differences were observed in general data such as age, sex, and disease duration between the two groups (*P >*0.05).

**Table 1:** Comparison of baseline clinical characteristics between the two groups.

Variable	Uncomplicated spinal tuberculosis group (n=194)	Neurological involvement group (n=100)	Test statistic	*P*-value
Age (years), M(Q_1_,Q_3_)	49.0 (33.0, 61.8)	50.5 (40.0, 65.3)	Z = -1.686	0.092
Sex, n(%)			χ² = 1.896	0.169
Female	82 (42.3)	35 (35.0)		
Male	112 (57.7)	65 (65.0)		
BMI (kg/m²), M(Q_1_,Q_3_)	22.4 (20.1, 24.6)	21.8 (19.5, 24.2)	Z = -1.245	0.213
Smoking History, n(%)	45 (23.2)	32 (32.0)	χ² = 2.714	0.099
Diabetes Mellitus History, n(%)	28 (14.4)	19 (19.0)	χ² = 1.034	0.309
Tuberculosis History, n(%)	15 (7.7)	8 (8.0)	χ² = 0.021	0.885
Symptom Duration (months), M(Q_1_,Q_3_)	4.0 (2.0, 8.0)	6.0 (3.0, 12.0)	Z = -2.834	0.005
Treatment Delay Time (months), M(Q_1_,Q_3_)	2.0 (1.0, 4.0)	3.0 (1.5, 6.0)	Z = -2.156	0.031

Comparative analysis of laboratory parameters (**Table 2**) demonstrated significant differences in several immune-inflammatory markers between the two groups. Specifically, patients with neurological involvement exhibited lower LYM and elevated MXD and PLR compared with those in the uncomplicated group (*P* < 0.05). No statistically significant differences were observed in WBC, CRP, or ESR.

**Table 2:** Comparison of laboratory parameters in 294 patients with spinal tuberculosis.

Variables	Total(n = 294)	Isolated spinal tuberculosis (n = 194)	Spinal tuberculosis with neurological involvement (n = 100)	Statistic	*P*
WBC, M (Q_1_, Q_3_)	6.290 (4.950, 7.500)	6.300 (4.990, 7.500)	6.260 (4.887, 7.470)	Z=-0.047	0.963
NEUT, M (Q_1_, Q_3_)	63.100 (55.950, 69.600)	62.150 (55.550, 68.375)	64.850 (58.000, 72.200)	Z=-2.020	0.043
LYM, M (Q_1_, Q_3_)	25.500 (19.200, 32.075)	26.150 (19.750, 32.900)	23.150 (16.900, 29.525)	Z=-2.689	0.007
MXD, M (Q_1_, Q_3_)	8.500 (7.000, 10.150)	8.100 (6.725, 9.900)	9.000 (7.375, 10.200)	Z=-2.370	0.018
EOS, M (Q_1_, Q_3_)	1.500 (1.000, 2.500)	1.500 (1.000, 2.500)	1.400 (0.800, 2.450)	Z=-1.038	0.299
NEUTcount, M (Q_1_, Q_3_)	3.780 (2.853, 4.938)	3.680 (2.798, 4.920)	4.025 (2.990, 5.022)	Z=-1.067	0.286
LYMcount, M (Q_1_, Q_3_)	1.455 (1.123, 1.880)	1.525 (1.170, 1.947)	1.365 (1.065, 1.830)	Z=-2.448	0.014
MXDcount, M (Q_1_, Q_3_)	0.525 (0.410, 0.680)	0.510 (0.390, 0.640)	0.575 (0.430, 0.690)	Z=-2.063	0.039
EOScount, M (Q_1_, Q_3_)	0.095 (0.060, 0.160)	0.100 (0.060, 0.160)	0.085 (0.060, 0.160)	Z=-1.022	0.307
RBC, M (Q_1_, Q_3_)	4.510 (4.152, 4.855)	4.510 (4.120, 4.882)	4.505 (4.178, 4.830)	Z=-0.163	0.871
HGB, M (Q_1_, Q_3_)	129.500 (118.000, 140.000)	129.000 (117.000, 139.750)	132.000 (119.000, 144.250)	Z=-0.577	0.564
PLT, M (Q_1_, Q_3_)	276.500 (220.000, 324.750)	278.000 (217.000, 327.000)	272.000 (230.750, 318.250)	Z=-0.036	0.971
PLR, M (Q_1_, Q_3_)	180.418 (131.797, 238.330)	174.980 (126.175, 228.304)	188.812 (151.964, 266.957)	Z=-2.667	0.008
CL, M (Q_1_, Q_3_)	105.400 (102.925, 107.700)	105.350 (103.025, 107.925)	105.400 (102.900, 107.375)	Z=-0.043	0.965
ALB, M (Q_1_, Q_3_)	37.100 (33.800, 39.475)	37.050 (33.900, 39.975)	37.350 (33.775, 39.125)	Z=-0.455	0.649
GLP, M (Q_1_, Q_3_)	33.200 (29.400, 36.975)	32.950 (29.125, 36.800)	33.200 (29.975, 37.325)	Z=-0.899	0.369
TC, M (Q_1_, Q_3_)	3.890 (3.330, 4.672)	3.970 (3.393, 4.698)	3.825 (3.308, 4.540)	Z=-0.997	0.319
AST, M (Q_1_, Q_3_)	18.300 (14.400, 26.200)	18.250 (14.075, 27.775)	18.300 (14.725, 24.800)	Z=-0.703	0.482
ALT, M (Q_1_, Q_3_)	16.300 (9.825, 25.925)	16.400 (9.850, 27.575)	16.200 (9.875, 23.925)	Z=-0.888	0.375
ALP, M (Q_1_, Q_3_)	92.000 (78.000, 110.000)	93.000 (78.000, 111.000)	91.000 (78.000, 110.000)	Z=-0.282	0.778
CRP, M (Q_1_, Q_3_)	18.650 (5.895, 35.675)	19.050 (5.355, 40.800)	17.950 (9.328, 30.825)	Z=-0.041	0.967
CAR, M (Q_1_, Q_3_)	0.504 (0.163, 1.054)	0.513 (0.148, 1.155)	0.484 (0.245, 0.912)	Z=-0.093	0.926
ESR, M (Q_1_, Q_3_)	38.000 (18.000, 59.000)	38.000 (17.000, 61.000)	37.000 (19.000, 54.000)	Z=-0.054	0.957

The distribution of ASIA neurological function grades in patients with spinal tuberculosis is detailed in **Table 3**, and the results of logistic regression analysis for factors influencing neurological involvement are presented in **Table 4**. Univariate analysis demonstrated that NEUT (odds ratio [OR]=1.025, 95% confidence interval [CI]: 1.001–1.051, *P* = 0.047), LYM (OR = 0.963, 95%CI: 0.936–0.990, *P* = 0.007), MXD (OR = 1.104, 95%CI: 1.012–1.204, *P* = 0.026), absolute LYMcount (OR = 0.569, 95%CI: 0.365–0.887, *P* = 0.013), and PLR (OR = 1.003, 95%CI: 1.001–1.005, *P* = 0.030) were significantly correlated with neurological involvement.

**Table 3:** Distribution of ASIA neurological function grades in patients with spinal tuberculosis.

ASIA Grade	Uncomplicated spinal tuberculosis group (n=194)	Neurological involvement group (n=100)	Total (n=294)
A	0	15 (15.0%)	15 (5.1%)
B	0	22 (22.0%)	22 (7.5%)
C	0	28 (28.0%)	28 (9.5%)
D	0	35 (35.0%)	35 (11.9%)
E	194 (100%)	0	194 (66.0%)

**Table 4:** Univariate and multivariate logistic regression analyses of factors influencing neurological involvement in patients with spinal tuberculosis.

Variables	Univariate analysis					Multivariate analysis				
	β	S.E	Z	*P*	OR (95%CI)	β	S.E	Z	*P*	OR (95%CI)
Sex										
0(female)					1.000 (Reference)					
1(male)	0.344	0.250	1.377	0.169	1.410 (0.864 ~ 2.300)					
Age	0.013	0.008	1.685	0.092	1.013 (0.998 ~ 1.028)					
WBC	0.022	0.060	0.373	0.709	1.023 (0.909 ~ 1.150)					
NEUT	0.025	0.012	1.990	**0.047**	1.025 (1.001 ~ 1.051)					
LYM	-0.038	0.014	-2.707	**0.007**	0.963 (0.936 ~ 0.990)	-0.039	0.014	-2.715	**0.007**	0.961 (0.935 ~ 0.989)
MXD	0.099	0.044	2.225	**0.026**	1.104 (1.012 ~ 1.204)	0.101	0.045	2.245	**0.025**	1.107 (1.013 ~ 1.209)
EOS	-0.069	0.074	-0.931	0.352	0.933 (0.807 ~ 1.079)					
NEUTcount	0.074	0.066	1.130	0.258	1.077 (0.947 ~ 1.226)					
LYMcount	-0.564	0.227	-2.489	**0.013**	0.569 (0.365 ~ 0.887)					
MXDcount	0.943	0.564	1.671	0.095	2.568 (0.850 ~ 7.760)					
EOScount	-0.929	1.168	-0.795	0.426	0.395 (0.040 ~ 3.896)					
RBC	0.070	0.222	0.314	0.754	1.072 (0.693 ~ 1.658)					
HGB	0.003	0.007	0.413	0.679	1.003 (0.989 ~ 1.016)					
PLT	0.000	0.001	0.098	0.922	1.000 (0.997 ~ 1.003)					
PLR	0.003	0.001	2.174	**0.030**	1.003 (1.001 ~ 1.005)					
CL	0.019	0.034	0.565	0.572	1.020 (0.953 ~ 1.090)					
ALB	-0.020	0.025	-0.775	0.439	0.981 (0.933 ~ 1.030)					
GLP	0.025	0.021	1.176	0.240	1.026 (0.983 ~ 1.069)					
TC	-0.112	0.117	-0.962	0.336	0.894 (0.711 ~ 1.123)					
AST	-0.013	0.009	-1.468	0.142	0.987 (0.971 ~ 1.004)					
ALT	-0.010	0.006	-1.538	0.124	0.990 (0.978 ~ 1.003)					
ALP	-0.002	0.003	-0.607	0.544	0.998 (0.992 ~ 1.004)					
CRP	-0.002	0.004	-0.467	0.641	0.998 (0.990 ~ 1.006)					
CAR	-0.030	0.122	-0.246	0.805	0.970 (0.763 ~ 1.233)					
ESR	-0.000	0.005	-0.050	0.960	1.000 (0.991 ~ 1.009)					

OR, Odds Ratio; CI, Confidence Interval. Meaning of the bold values: Univariate analysis showed that NEUT, LYM, MXD, LYMcount, and PLR were significantly associated with neurological involvement (all P < 0.05). After adjustment for inter-variable confounding by multivariate Logistic regression (forward stepwise), only LYM (OR = 0.961, P = 0.007) and MXD (OR = 1.107, P = 0.025) were identified as independent influencing factors, suggesting that the univariate associations of other variables may be mediated by collinearity and overall immunoinflammatory effects.

These variables were further incorporated into a multivariate logistic regression model, and stepwise forward selection was applied for variable screening. The results identified that LYM (β=-0.039, standard error [SE]=0.014, Z=-2.715, *P* = 0.007) and MXD (β=0.101, SE = 0.045, Z = 2.245, *P* = 0.025) were independent influencing factors for neurological involvement in patients with spinal tuberculosis. Multivariate analysis revealed that for every 1-percentage-point decrease in LYM, the risk of neurological involvement increased by approximately 3.9%; for every 1% increase in MXD, the risk of neurological involvement increased by 10.7% (OR = 1.107, 95%CI: 1.013–1.209). The Hosmer-Lemeshow test confirmed a good model fit (χ²=4.215, *P* = 0.837).

### 3.1 Subgroup analysis according to ASIA severity

Within the neurological involvement group, patients were further stratified into severe neurological impairment (ASIA grades A–B) and mild-to-moderate impairment (ASIA grades C–D).

Comparative analysis showed that patients with more severe neurological impairment tended to have lower lymphocyte percentage (LYM) and higher mixed cell percentage (MXD) compared with those with milder impairment. However, the differences did not reach statistical significance.

These findings suggest a potential association between immune-inflammatory imbalance and the severity of neurological injury, although further studies with larger sample sizes are required to confirm this relationship.

Multicollinearity diagnostics showed that the VIF values for LYM and MXD were both below the threshold of 5, indicating no significant collinearity between the variables and confirming the stability of the regression model.

### 3.2 Calibration curve

**Figure 2:**
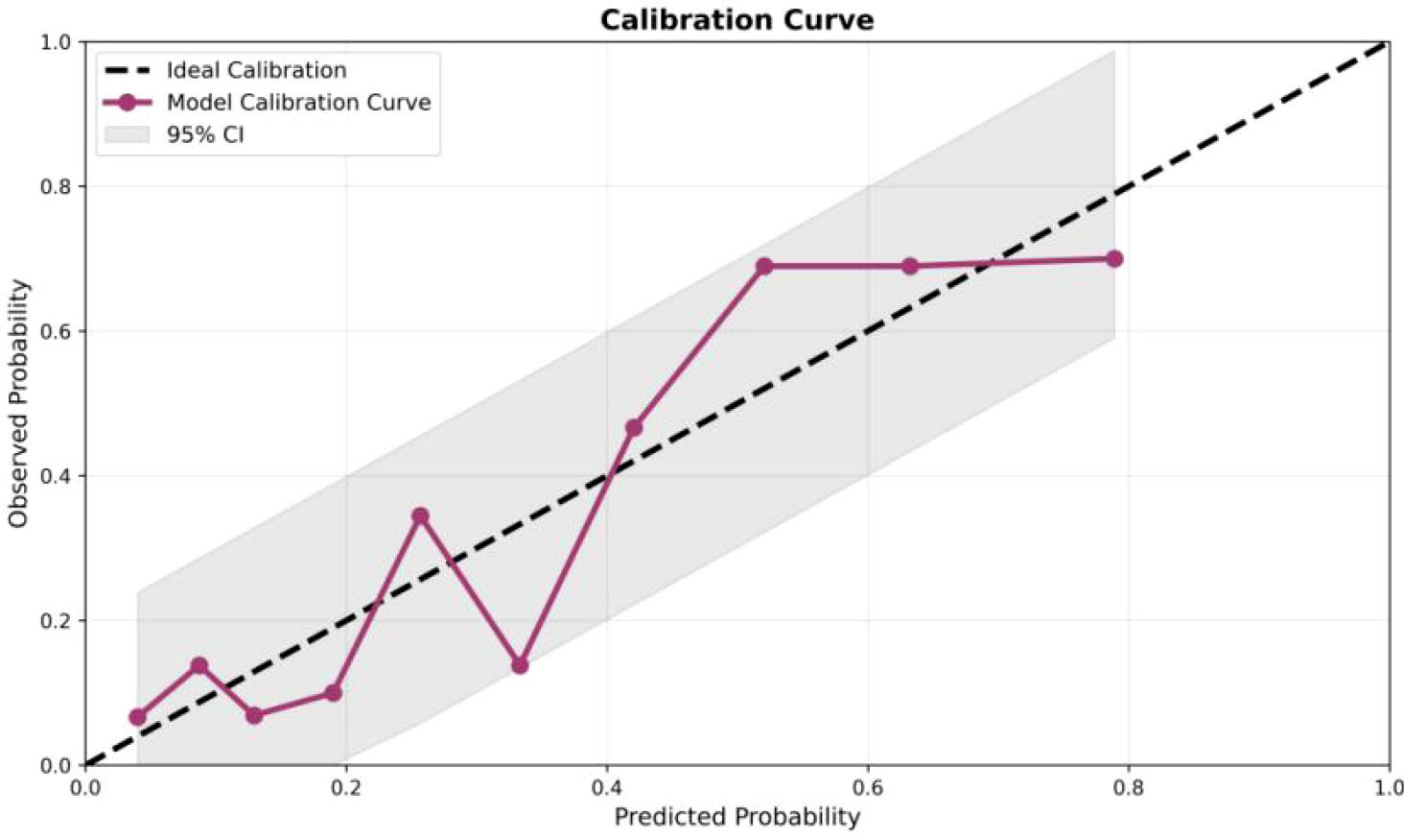
Calibration curve.

To evaluate the consistency between the predicted probabilities of the model and the actual observed risks, we plotted a calibration curve and performed the Hosmer-Lemeshow goodness-of-fit test. The results showed that the calibration curve was highly consistent with the ideal 45° reference line (slope = 1.02, intercept = -0.01), indicating minimal systematic error across the entire risk spectrum (**Figure 2**). Patients were stratified into 10 risk groups based on their predicted probabilities for the Hosmer-Lemeshow test, yielding a χ² statistic of 4.215 (degrees of freedom = 8, *P* = 0.837), which demonstrated no significant discrepancy between the predicted and observed values. The Brier score was 0.185, comprehensively reflecting favorable discrimination and calibration performance.

Visualization analysis revealed that the calibration curve closely approximated the ideal line across the predicted probability range of 0.1–0.6, with a narrow 95% confidence interval band, confirming the stable performance of the model across low-, moderate-, and high-risk subgroups. Notably, the model maintained robust calibration at the critical clinical decision threshold (0.3–0.4), providing a reliable basis for determining the optimal timing of intervention. Collectively, these findings verify that the multivariate model constructed based on LYM and MXD can generate robust probability estimates that accurately reflect the actual risk of neurological complications, supporting its clinical application for patient stratification management and early surgical decision-making. Despite the excellent calibration of the model, prospective external validation studies are still required to confirm its generalizability across diverse populations.

**Figure 3:**
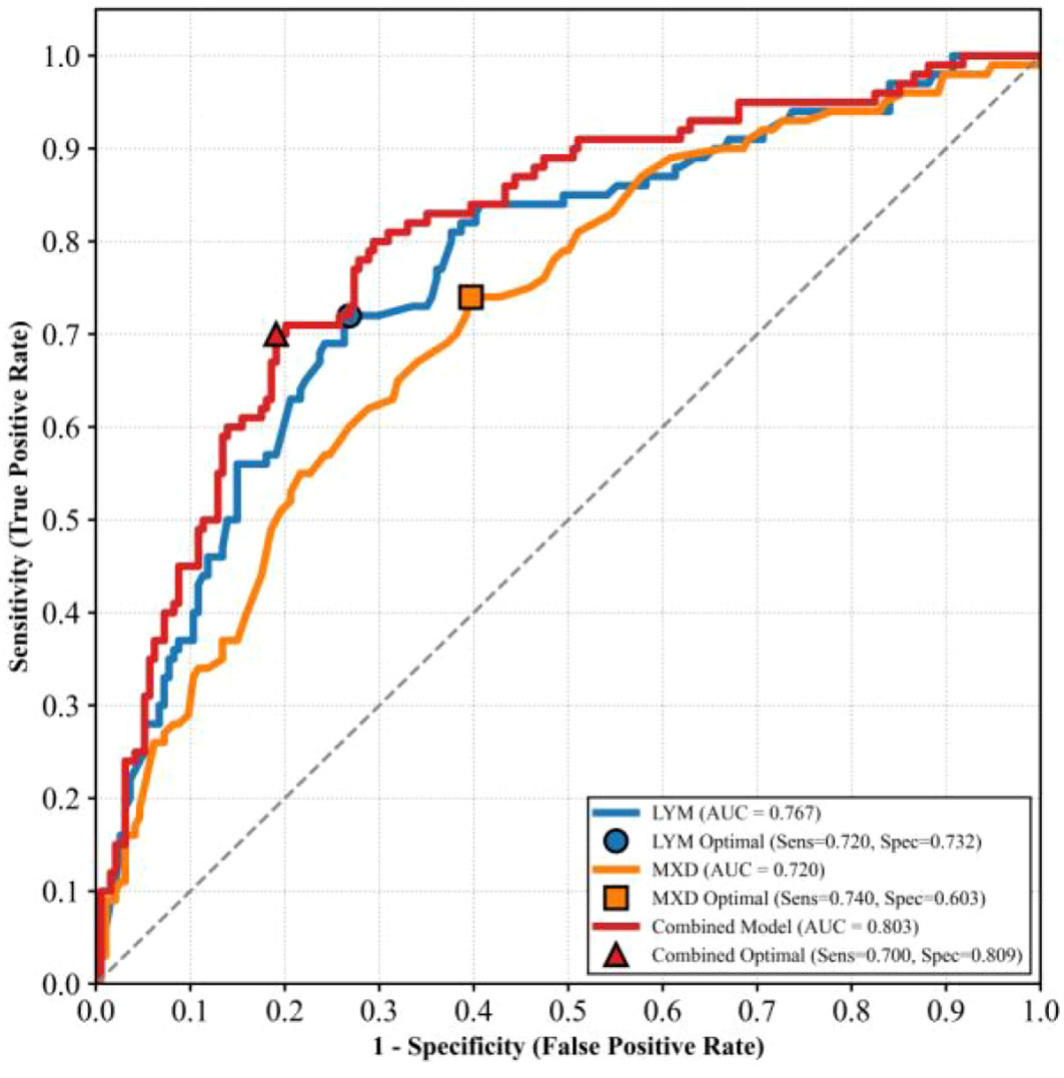
ROC curve analysis of LYM, MXD and their combined model for predicting neurological involvement.

To further evaluate the clinical predictive value of the aforementioned indicators, we plotted receiver operating characteristic (ROC) curves to analyze their diagnostic efficacy (**Figure 3**, **Table 5**). The results showed that the area under the curve (AUC) for LYM alone was 0.767 (95% confidence interval [CI]: 0.711–0.828), with an optimal cutoff value of 23.7%, a sensitivity of 72.0%, and a specificity of 73.2%. The AUC for MXD alone was 0.720 (95%CI: 0.658–0.780), with an optimal cutoff value of 8.2%, a sensitivity of 74.0%, and a specificity of 60.3%.

**Table 5:** Statistical values of ROC curve analysis for LYM, MXD and their combined model in predicting neurological involvement.

Indicator	AUC	95%CI	Cut off	Sensitivity	Specificity	Youden index
LYM	0.767	0.711-0.828	23.7%	72.0%	73.2%	0.452
MXD	0.720	0.658-0.780	8.2%	74.0%	60.3%	0.343
Combined	0.803	0.749-0.857	0.41%	70.0%	80.9%	0.509

After constructing a combined prediction model incorporating these two indicators, the AUC was improved to 0.803 (95%CI: 0.749–0.857), with a sensitivity of 70.0%, a specificity of 80.9%, and a Youden’s index of 0.509. This combined model exhibits higher predictive accuracy than either single indicator, and thus can serve as an objective tool for the early clinical identification of high-risk patients with neurological involvement.

**Figure 4:**
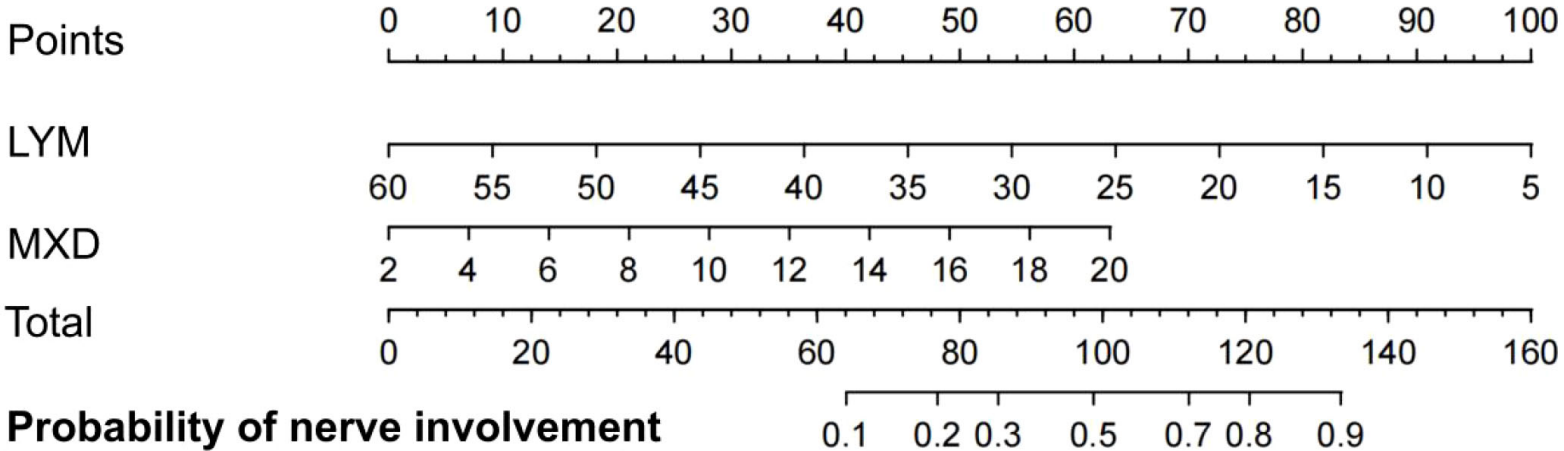
Nomogram.

Based on the multivariate logistic regression model, this study constructed a nomogram for predicting the risk of neurological involvement in patients with spinal tuberculosis (**Figure 4**). This nomogram incorporated two independent influencing factors, namely LYM and MXD, and converted the complex regression equation into a visualized clinical tool.

For application, the actual measured values were located on the variable axes corresponding to LYM and MXD according to the results of the first fasting complete blood count after the patient’s admission. Vertical lines were drawn upward to determine the individual scores, and the total score was obtained by summing the two individual scores. Subsequently, the total score was located on the total score axis, and a vertical line was drawn downward to read the predicted probability of neurological involvement in the patient. For instance, an LYM of 20% corresponds to approximately 32 points, an MXD of 10% corresponds to approximately 28 points, and a total score of 60 points corresponds to a neurological involvement risk of approximately 65%.

This nomogram features simple operation without the need for complex calculations, enabling intuitive and rapid individualized risk assessment. It helps clinicians identify high-risk patients at the bedside or in outpatient clinics at an early stage, and provides an objective and quantitative reference basis for optimizing the timing of imaging examinations, formulating surgical decisions, and implementing intensive anti-tuberculosis therapy.

## 4 Discussion

This study identified LYM and MXD as independent predictors of neurological involvement in spinal tuberculosis, based on multivariate analysis. It was further transformed into a visualized nomogram tool, enabling the clinical operationalization of individualized risk assessment. Although LYM and MXD are both derived from the leukocyte differential count, multicollinearity analysis using VIF indicated acceptable independence between these variables, supporting the robustness of the model. Both the Hosmer-Lemeshow test (χ²=4.215, *P* = 0.837) and calibration curve analysis confirmed the excellent calibration of the model, with predicted probabilities highly consistent with actual observed risks. This provides a simple, cost-effective, and reliable quantitative tool for the early clinical identification of high-risk patients.

The pathological mechanisms underlying neurological impairment complicating spinal tuberculosis involve multiple factors, such as mechanical compression caused by vertebral collapse, spinal cord edema, ischemia-reperfusion injury, and direct invasion by Mycobacterium tuberculosis (Xie et al., 2025; Le et al., 2025). Space-occupying lesions including paravertebral abscesses not only induce neurological involvement through direct mechanical compression but also may further exacerbate spinal cord injury by triggering intense inflammatory responses. During abscess formation, a large number of local and systemic pro-inflammatory mediators are released, activating the innate immune system and suppressing cellular immune function, which is manifested as a significant decrease in peripheral blood LYM and a compensatory increase in MXD. This immune imbalance may synergize with mechanical compression to promote the occurrence and progression of neurological dysfunction through pathways such as disrupting the blood-spinal cord barrier, aggravating spinal cord edema and ischemia-reperfusion injury (Pi et al., 2024; Rahyussalim et al., 2023; Wu et al., 2023). Therefore, LYM and MXD are not only passive markers of systemic inflammation but may also actively participate in the pathological process of nerve injury, providing a reasonable pathophysiological explanation for their role as independent predictors. Existing studies have mostly focused on local anatomical changes, such as imaging parameters including spinal canal encroachment rate, kyphotic Cobb angle, and abscess volume (Li et al., 2023; Shen et al., 2021), while the role of the host’s systemic immune-inflammatory response in the progression of nerve injury has not received sufficient attention. In recent years, the value of peripheral blood inflammatory indices in tumor prognosis evaluation, sepsis severity stratification, and cardiovascular event prediction has been widely confirmed (Tan et al., 2024; Evans et al., 2023; Ang et al., 2025; Wang et al., 2024). As core effector cells of cellular immunity, the reduced LYM reflects the immunosuppressive state induced by tuberculosis infection, which may exacerbate the local inflammatory cascade in the spinal cord by impairing the ability to clear Mycobacterium tuberculosis (Lukeman et al., 2025; Musvosvi et al., 2023). The elevated MXD (including monocytes, basophils, etc.) indicates the sustained activation of the innate immune system, and the pro-inflammatory cytokines released by these cells can disrupt the blood-spinal cord barrier and aggravate spinal cord edema and ischemic injury (Li et al., 2025; Franken et al., 2022). In addition, the PLR, as a comprehensive marker of systemic inflammation, was also found to be significantly correlated with neurological involvement in the univariate analysis of this study, but its effect was explained by LYM and MXD in the multivariate model. Notably, traditional inflammatory indices such as CRP and ESR showed no significant differences between the two groups in this study, which may be related to their delayed response and insufficient specificity in tuberculosis infection, also highlighting the potential advantages of novel immune-inflammatory indices. Although foreign studies have reported the correlation between lymphocyte subsets and the severity of tuberculosis (Guo et al., 2025; Woodworth et al., 2021; Simonson et al., 2025), specific prediction models for neurological involvement in spinal tuberculosis remain scarce, and this study fills this key gap in the field.

From a clinical perspective, the investigated immune-inflammatory markers may provide valuable information for the early identification of patients at risk of neurological involvement. Unlike imaging findings, which often reflect already established structural damage, changes in peripheral blood markers may occur earlier in the disease course. Therefore, LYM and MXD could serve as accessible early warning indicators, particularly in settings where advanced imaging is not readily available.

The clinical significance of this study is mainly reflected in four aspects. First, this study is the first to confirm that peripheral blood LYM and MXD are independent risk factors for neurological involvement in spinal tuberculosis, providing a new perspective for understanding the immune pathological mechanism of tuberculous spinal cord injury. Traditional concepts have mainly focused on local mechanical compression, while this study reveals the driving role of the host immune status in neurological impairment, emphasizing the importance of individualized immune assessment. Second, the constructed combined prediction model can complete risk assessment using only two routine blood parameters, without the need for additional expensive examinations. It has excellent accessibility and promotion value in primary medical institutions, helping to narrow the gap in diagnosis and treatment levels among different regions. Third, the development of the nomogram realizes the bedside translation of complex statistical models. Clinicians can quickly calculate individualized risk probabilities, providing an objective basis for optimizing the timing of imaging examinations, formulating surgical decisions, and implementing intensive anti-tuberculosis therapy. It is particularly suitable for identifying occult nerve injuries with atypical early symptoms. Fourth, the model maintains stable calibration at the critical clinical decision threshold (0.3–0.4), providing reliable support for the selection of intervention timing, and helping to avoid the dual risks of overtreatment and delayed treatment. Compared with the lag of traditional ASIA grading evaluation, this model provides a prospective risk early warning paradigm, which is in line with the development direction of precision medicine.

This study has several limitations that need to be addressed in subsequent research. First, although the single-center retrospective study design ensures the convenience of data collection, it may be subject to selection bias. All study subjects are from the Ningxia region, and their demographic characteristics, Mycobacterium tuberculosis strain types, and genetic backgrounds may limit the generalization ability of the model in populations from different geographical areas. Multicenter, prospective external validation is urgently needed to evaluate the generalizability and robustness of the model. Second, the inclusion criteria of the study are limited to patients who underwent surgical treatment, excluding critically ill cases receiving conservative treatment or those unable to tolerate surgery, which may lead to insufficient sample representativeness. Future studies should cover a broader disease spectrum to improve the applicability of the model. Third, the study did not include local anatomical variables such as imaging parameters including spinal canal encroachment degree, abscess volume, and kyphotic deformity angle. Although this highlights the independent value of immune indices, it may ignore the interaction between imaging and immune indices. Combined modeling is expected to further improve the predictive efficacy. Finally, although the sample size meets the needs of the current analysis, the statistical power for rare subgroups (such as ASIA grade A complete injury) is still insufficient. The accumulation of large cohorts will facilitate the development of subgroup-specific models. Future research can also explore the application potential of machine learning algorithms in constructing nonlinear prediction models, and conduct randomized controlled trials to verify whether the risk-stratified intervention strategy based on this model can effectively improve patient prognosis.

It should be noted that neurological involvement was defined as a composite outcome (ASIA grades A–D) in this study. However, substantial heterogeneity exists across different ASIA grades in terms of clinical severity and prognosis.

Our exploratory subgroup analysis suggested a potential relationship between immune-inflammatory markers and the severity of neurological impairment, although statistical power was limited. Future studies should aim to develop severity-specific prediction models to better support clinical decision-making.

## 5 Conclusion

This study confirms that decreased peripheral blood LYM and increased MXD are independent risk factors for neurological involvement in patients with spinal tuberculosis. The combined prediction model constructed based on these two indicators exhibits favorable diagnostic efficacy (AUC = 0.803) and good calibration.

Featuring simple operation, cost-effectiveness and practicality, this model is further transformed into a nomogram that enables the early identification of high-risk patients, thereby providing an objective and quantitative tool for clinical individualized treatment decision-making. Despite limitations including the single-center retrospective study design, this research still plays an important foundation for establishing an early warning system for neurological complications of spinal tuberculosis. Multicenter prospective validation is required in future studies to further optimize and promote the application of this model.

## Data Availability

The raw data supporting the conclusions of this article will be made available by the authors, without undue reservation.
